# 3D-Printed Patient-Specific ACL Femoral Tunnel Guide from MRI

**DOI:** 10.2174/1874325001812010059

**Published:** 2018-02-28

**Authors:** Iain Rankin, Haroon Rehman, Mark Frame

**Affiliations:** 1 Aberdeen Royal Infirmary - Trauma and Orthopaedic Surgery, Foresterhill Aberdeen AB25 2ZN, United Kingdom of Great Britain and Northern Ireland; 2 University Hospital Southampton NHS Foundation Trust Ringgold standard institution - Trauma and Orthopaedic Surgery Southampton, Southampton, United Kingdom of Great Britain and Northern Ireland

**Keywords:** Anterior Cruciate Ligament, ACL, Anatomic, Anterior Cruciate Ligament reconstruction, Printing, Three-dimensional, Arthroscopy

## Abstract

**Background:**

Traditional ACL reconstruction with non-anatomic techniques can demonstrate unsatisfactory long-term outcomes with regards instability and the degenerative knee changes observed with these results. Anatomic ACL reconstruction attempts to closely reproduce the patient's individual anatomic characteristics with the aim of restoring knee kinematics, in order to improve patient short and long-term outcomes. We designed an arthroscopic, patient-specific, ACL femoral tunnel guide to aid anatomical placement of the ACL graft within the femoral tunnel.

**Methods:**

The guide design was based on MRI scan of the subject's uninjured contralateral knee, identifying the femoral footprint and its anatomical position relative to the borders of the femoral articular cartilage. Image processing software was used to create a 3D computer aided design which was subsequently exported to a 3D-printing service.

**Results:**

Transparent acrylic based photopolymer, PA220 plastic and 316L stainless steel patient-specific ACL femoral tunnel guides were created; the models produced were accurate with no statistical difference in size and positioning of the center of the ACL femoral footprint guide to MRI (*p*=0.344, *p*=0.189, *p*=0.233 respectively). The guides aim to provide accurate marking of the starting point of the femoral tunnel in arthroscopic ACL reconstruction.

**Conclusion:**

This study serves as a proof of concept for the accurate creation of 3D-printed patient-specific guides for the anatomical placement of the femoral tunnel during ACL reconstruction.

## 1 INTRODUCTION

Anterior cruciate ligament (ACL) reconstruction has repeatedly demonstrated successful outcomes at short term follow-up. It aims to improve the stability of the knee, facilitate return to sports and may help prevent osteoarthritis produced in ACL deficient knees [1-3]. However, several studies can demonstrate unsatisfactory long-term outcomes, particularly in high level athletes, owing to the development of clinically symptomatic instability and a low rate of return to pre-injury sporting levels [4-9].

Traditional ACL reconstruction included transtibial drilling of the ACL femoral tunnel, with a focus on isometric graft placement and avoidance of notch impingement [10]. More recently, surgical techniques for creating the ACL femoral tunnel have been reconsidered, with a focus towards anatomical placement [11-15]. Anatomic ACL reconstruction can be defined as the functional restoration of the ACL to its native dimensions, collagen orientation and insertion sites [16]. Femoral tunnel anatomical positioning is achieved through marking of the tunnel via the anteromedial or an accessory anteromedial portal [12-16]. Retrograde reamers have also been introduced as a method to aid in placement of the femoral tunnel at the anatomical ACL position [17]. A primary focus towards anatomic reconstruction has been shown to better restore anterior translational as well as rotational stability to an ACL deficient knee [18-21].

The ACL ‘femoral footprint’ is described as the midpoint between the anteromedial and posterolateral ACL bundles - marking the midpoint of the native anatomical ACL [22]. The mean anatomic centrum of the ACL femoral footprint has been described radiologically as 43% of the distance from the proximal margin of the posterior condyle of the femur to the distal most aspect of the condyle, as viewed on a lateral radiograph of the lateral wall of the intercondylar notch [23]. Marking the femoral footprint through direct visualization arthroscopically has been described through identifying the lateral intercondylar ridge and marking a point half way between this and the inferior articular cartilage [24]. Whilst these measurements and landmarks provide a reference, they are not specific to any one patient’s anatomy. Non-anatomic ACL graft placement is the most common technical error leading to recurrent instability following ACL reconstruction [25, 26]. Mid-bundle techniques potentially have a higher graft re-rupture rate, however, this does not take into account truly anatomical placement of the graft in accordance with the patient’s native femoral footprint as identified on MRI. Patient specific ACL reconstruction has been proposed as a means to achieving a truly anatomical reconstruction [27].

Three-dimensional (3D) printed guides have been reported for various orthopaedic procedures, such as pelvic osteotomy [28], fixation for acetabular fracture [29], spinal instrumentation [28, 30], knee arthroplasty [31], hip arthroplasty [32] and corrective osteotomy of the upper extremity [33, 34].

Our aim was to design a 3D printed patient specific ACL femoral tunnel guide, based on magnetic resonance imaging (MRI) scan of the patient’s contralateral uninjured knee, for accurate intraoperative placement of the femoral tunnel within the ACL femoral footprint in single bundle ACL reconstruction to place the femoral tunnel in a truly anatomical position.

## 2 METHODS

A standard protocol MRI of a patient’s knee without ACL injury was carried out. The scanners used were 1.5 Tesla Siemens scanners. We used a MRI protocol for fat-saturated proton density images (PDFS) in 3 planes, coronal/axial/sagittal, and a sagittal T1 weighted image.

**Fig. (1):**
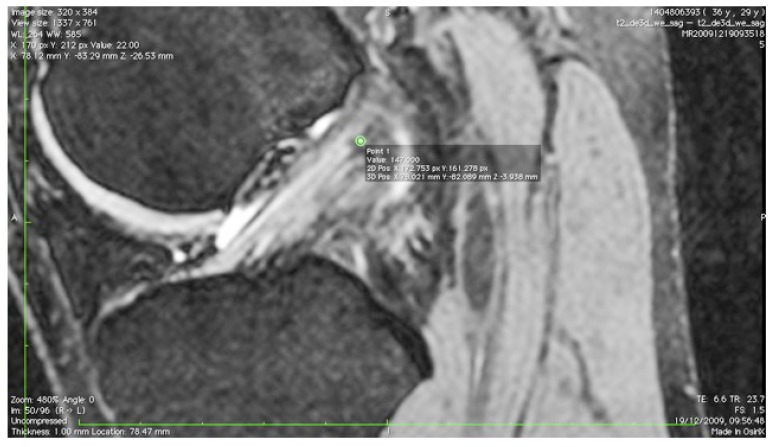
The patient’s native ACL femoral footprint (represented as point 1).

**Fig. (2):**
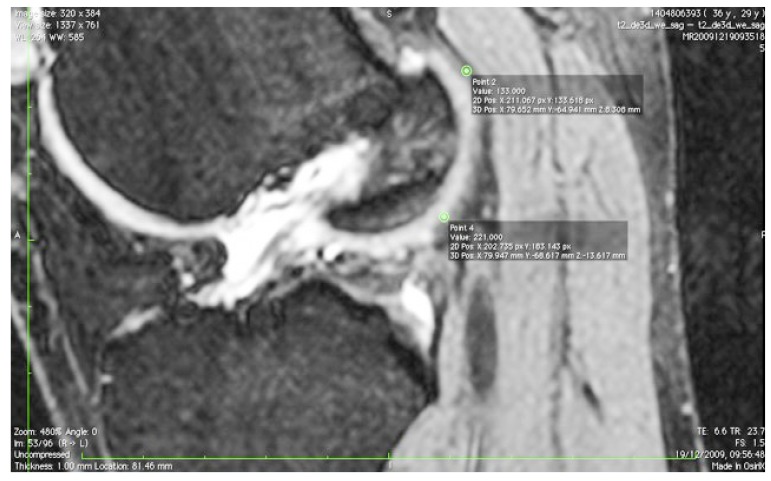
The proximal and posterior edge of the articular cartilage on the lateral wall of the femoral notch (represented as points 2 and 4 respectively).

**Fig. (3):**
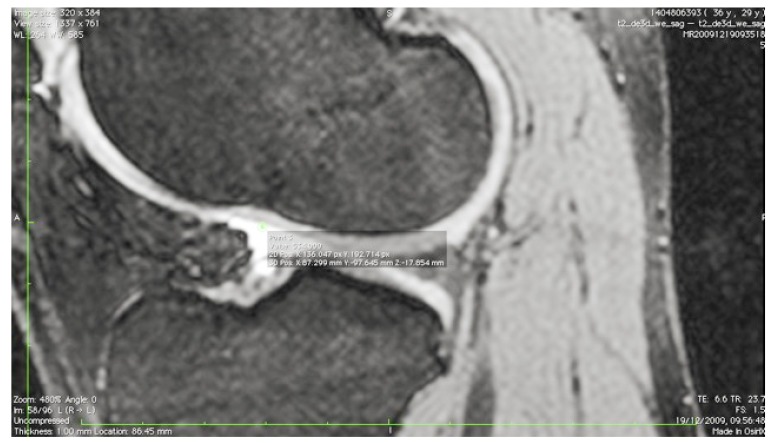
The distal edge of the articular cartilage on the lateral wall of the femoral notch (represented as point 3).

**Fig. (4):**
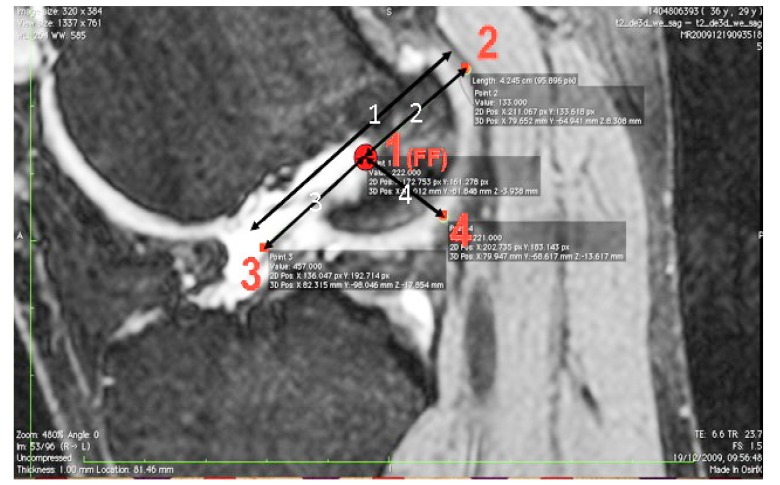
Annotated MRI highlighting the anatomical landmarks analyzed (marked in red): the patient’s native ACL femoral footprint (1) and the proximal (2), distal (3), and posterior (4) edge of the articular cartilage on the lateral wall of the femoral notch. The annotated black arrows with white numbers represent the distances determined for creation of the ACL guide: total guide internal length (proximal to distal femoral cartilage points, distance 1) and the position of the native ACL femoral footprint relative to the three articular cartilage points (distances 2, 3 and 4). Red point 1 (FF): Femoral footprint. Red point 2: Proximal edge of articular cartilage. Red point 3: Distal edge of articular cartilage. Red point 4: Posterior edge of articular cartilage. Distance 1: Proximal – distal. Distance 2: Proximal – femoral footprint. Distance 3: Distal – femoral footprint. Distance 4: Posterior – femoral footprint.

The images were transferred *via* DICOM files to a personal computer running OsiriX image processing software (Pixmeo, Geneva, Switzerland). Images were then subsequently analyzed for several anatomical landmarks: the patient’s native ACL femoral footprint (Fig. **1**), the proximal and posterior edges (Fig. **2**), and the distal edge (Fig. **3**) of the articular cartilage on the lateral wall of the femoral notch. Distances were then calculated to determine the position of the center of the ACL footprint relative to the three articular cartilage points (Fig. **4**). Three independent trained observers (orthopaedic surgeons) carried out three separate measurements for each anatomical landmark. The mean of the multiple measurements for each landmark was then used for subsequent analysis and 3D printing. Inter-observer variation was measured by means of intraclass correlation coefficient (ICC) with 95% confidence intervals (CI) using a random-effects model.

These measurements and points were then utilized to create a 3D computer aided design (CAD) model of a custom guide. This was done using the 3D CAD program 123Design (Autodesk Ltd., Farnbourgh, UK). The guides were designed with an entry point at the site of the ACL femoral footprint to allow access of a 3mm Chondro Pick (Arthrex inc., Naples, Florida) through the guide to mark the starting point of the femoral tunnel. The 3D model was exported as an STL file suitable for 3D printing. The STL file was uploaded to an online 3D printing service and the physical guide was created in transparent acrylic based photopolymer, PA220 plastic and 316L stainless steel.

The models created were measured using vernier calipers (Mitutoyo 500-196-20 0-150 mm 6-inch Absolute Digimatic Caliper, Mitutoyo Corp., Japan). Three independent observers carried out three separate measurements of the models. The mean of the multiple measurements was compared to the original MRI dimensions and 3D CAD model (Graphpad Prism 6, Graphpad Inc. CA, USA). Paired student t test was performed to assess for statistical significance. Inter-observer variation was measured by means of ICC with 95% CI using a random effects model.

## 3 RESULTS

**Fig. (5):**
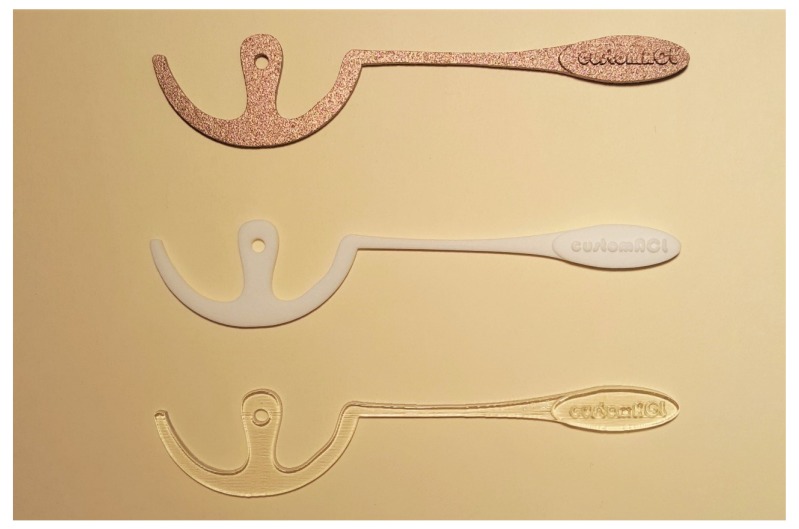
Three patient specific ACL femoral tunnel guides: 316L stainless steel, PA220 plastic and transparent acrylic based photopolymer.

Three patient specific ACL femoral tunnel guides (transparent acrylic based photopolymer, PA220 plastic and 316L stainless steel) were created. (Fig. **5**) Distances measured included proximal to distal articular cartilage, posterior articular cartilage to femoral footprint, distal articular cartilage to femoral footprint and proximal articular cartilage to femoral footprint. The models produced were accurate with no statistical difference in size and positioning of the center of the ACL femoral footprint, relative to the articular cartilage margins on the lateral wall of the femoral notch, when compared to the original CAD model and MRI scans (MRI/CAD *Vs*. PA220 *p*=0.3753, MRI/CAD *Vs*. 316L *p*=0.0683, MRI/CAD *Vs*. Photopolymer *p*=0.3450) (Table **1**). Inter-observer variability analysis showed excellent correlation for both MRI landmark identification (ICC 1.00, CI 0.997 to 1.000) and guide measurement (ICC 1.00, CI 1.00 to 1.00). The costs for the 3D printed models were £3.50 for the PA220 plastic, £15 for the transparent photopolymer and £25 for the 316L stainless steel. The time taken from MRI to delivery for the physical models was 7 days.

## 4 DISCUSSION

Our study demonstrates that a 3D printed patient-specific ACL femoral tunnel guide can be created, based on a MRI scan of the contralateral uninjured knee, with low cost and of short duration from conception to creation. The guide, *via* entry of the anterolateral portal, would allow the operating surgeon to mark out the starting point of the femoral tunnel with a 3mm Chondro Pick, via the entry point within the guide.

There is increasing evidence indicating that the anatomic ACL reconstruction produces greater restoration of anterior translational as well as rotational stability to an ACL deficient knee [18-21]. Anatomic reconstruction of the ACL should take into account the differences between the anatomical characteristics of each patient in order to potentially restore native ligament function, with known variation between individuals in the shape and size of the ACL [35]. A “one-size-fits-all” approach does not adequately reproduce the native ACL. Non-anatomical reconstruction procedures may eliminate anterior/posterior laxity, but fail to restore rotational stability [36, 37]. This has been investigated with *In vivo* kinematic studies, which showed that non-anatomic ACL reconstruction procedures fail to restore normal dynamic knee function. Georgoulis *et al*. examined ACL-deficient knees before and after non-anatomic ACL reconstruction, using video-motion analysis. ACL-deficient patients demonstrated greater tibial internal rotation during walking. This reached near normal levels following non-anatomical reconstruction. During higher demand activities however, such as stair descent and pivoting, tibial rotation was significantly larger in the non-anatomical ACL reconstructed knees compared to the contralateral intact ACL knee [38]. Brandsson *et al*. similarly found that tibial rotation was not restored with non-anatomical ACL reconstruction when measured using continuous radiostereometric analysis [39]. MRI investigation during static weightbearing showed that whilst non-anatomical ACL reconstruction reduced sagittal laxity of the knee to within normal limits, it did not restore the normal tibiofemoral kinematics [40]. More physically demanding activities have been investigated using high-speed radiographic imaging systems. Tashman *et al*. used a 250 frame/s dynamic stereo x-ray system to evaluate *in vivo* kinematic of the knee during downhill running in patients that had undergone non-anatomic ACL reconstruction. Anteroposterior translation was restored, but the reconstructed knees were more externally rotated and more adducted relative to the contralateral, uninjured knees. These rotational changes were associated with shifts in the areas of joint contact and a reduction in medial-compartment joint space during dynamic loading [41, 42]. Standard non-anatomical tunnels reproduce only a fraction of the native ACL. With respect to the femoral insertion, Hensler *et al*. showed that only 61% of the femoral insertion is reconstructed with standard tunnel preparation [43]. Abebe *et al*., using biplanar fluoroscopy and MRI, reported that anatomic femoral placement of the graft resulted in kinematics that more closely replicated that of the intact knee when compared to a non-anatomical femoral placement [44]. By individualizing ACL reconstruction, we may be able to reproduce more of the native anatomy and improve patient outcomes.

Articles regarding the creation of a 3D printed custom ACL guide from the patient’s contralateral knee do not feature in current literature. Where 3D printed patient specific guides have been used in other orthopedic procedures, favorable outcomes have been achieved. Reported advantages to patient-specific surgical guides include a reduction in operating times and improvement in the accuracy of surgical interventions, due to the guides’ personalization [45]. Hananouchi *et al* developed a 3D printed patient specific surgical guide for cup insertion in total hip arthroplasty. In their study, the mean absolute deviation from the patient’s preoperative planned alignment of the cup was 2.8**°** for abduction and 3.7**°** for anteversion [32]. A cadaveric study utilizing customized cutting jigs for total knee arthroplasty showed mean errors for alignment and bone resection within 1.7**°** and 0.8 mm respectively [30]. Whilst showing desirable results, the authors of these studies did not compare the results of customized guides to outcomes achieved without customized guides. Patient specific guides have increased popularity in spinal surgery owing to the reported improvement in accuracy of instrumentation [45]. Bundoc *et al*. developed a 3D printed patient specific drill guide for pedicle screw insertion into the subaxial cervical spine. They performed a cadaveric study on fifty pedicles to investigate the accuracy of screw placement. Their findings showed the patient specific guides to have an overall accuracy rate for cervical pedical screw placement of 94%, greater than the current reported gold standard (fluoroscopy-guided insertion) accuracy rates of 85-91% [46]

In the case of total knee arthroplasty, a recent meta-analysis has shown that patient specific guides provide no superior accuracy than using manual implementation during total knee arthroplasty [47]. There is no data regarding outcomes of patient-specific ACL guides currently within the literature.

It is hypothesized that the use of a patient specific guide would allow better identification of the ACL footprint than notch clearance and visualization of the ACL alone. A clear ACL footprint within the injured knee is not always available. Identification of the exact origins of the ACL in a traumatic knee as viewed through a 30-degree arthroscope is not always possible. The next step for this research is a cadaveric based study, utilizing the guides to carry out the creation of the ACL femoral tunnel and subsequent analysis of tunnel placement in relation to the contralateral knee.

The guides were easy to create and produce, taking only a week and with a cost of between £3.50 and £25. A modified MRI protocol scanning both knees of patients with suspected ACL injuries would allow identification of the native ACL femoral footprint at the same time of diagnosis of ACL injury, MRI of the contralateral knee utilizing only the 2 planes required for guide design incurs an additional ten minutes of MRI time. Following sterilization, these surgical guides could be used intraoperatively in any hospital.

This study serves as the first step and a proof of concept for the accurate creation of patient specific 3D printed guides for the anatomical placement of the femoral tunnel during ACL reconstruction.

## Figures and Tables

**Table 1:**
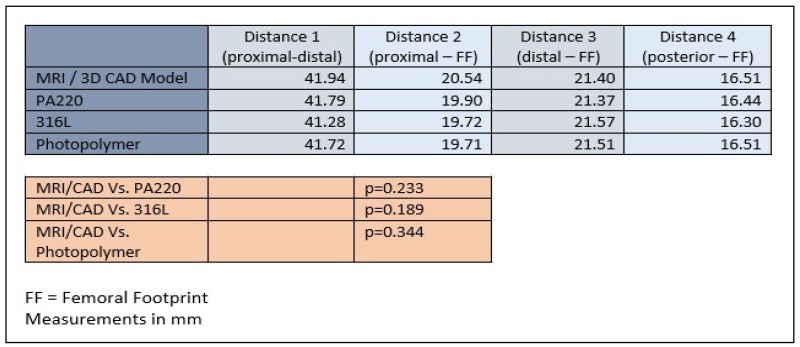
Mean measurements of patient specific ACL femoral tunnel guides. Distances measured included proximal to distal articular cartilage (distance 1), posterior articular cartilage to femoral footprint (distance 2), distal articular cartilage to femoral footprint (distance 3) and proximal articular cartilage to femoral footprint (distance 4). No statistical significant differences were found in the size of models or the position of the femoral footprint when compared to the computer assisted design or patient MRI. FF = Femoral Footprint. Measurements in mm.

## References

[1] Jomha N.M., Pinczewski L.A., Clingeleffer A., Otto D.D. (1999). Arthroscopic reconstruction of the anterior cruciate ligament with patellar-tendon autograft and interference screw fixation. The results at seven years.. J. Bone Joint Surg. Br..

[2] Marcacci M., Zaffagnini S., Iacono F., Vascellari A., Loreti I., Kon E., Presti M.L. (2003). Intra and extra-articular anterior cruciate ligament reconstruction utilizing autogeneous semitendinosus and gracilis tendons: 5-year clinical results.. Knee Surg. Sports Traumatol. Arthrosc..

[3] Giron F., Aglietti P., Cuomo P., Mondanelli N., Ciardullo A. (2005). Anterior cruciate ligament reconstruction with double-looped semitendinosus and gracilis tendon graft directly fixed to cortical bone: 5-year results.. Knee Surg. Sports Traumatol. Arthrosc..

[4] Lewis P.B., Parameswaran A.D., Rue J.P., Bach B.R. (2008). Systematic review of single-bundle anterior cruciate ligament reconstruction outcomes: A baseline assessment for consideration of double-bundle techniques.. Am. J. Sports Med..

[5] Ardern C.L., Taylor N.F., Feller J.A., Webster K.E. (2012). Return-to-sport outcomes at 2 to 7 years after anterior cruciate ligament reconstruction surgery.. Am. J. Sports Med..

[6] Shelbourne K.D., Gray T. (2000). Results of anterior cruciate ligament reconstruction based on meniscus and articular cartilage status at the time of surgery. Five- to fifteen-year evaluations.. Am. J. Sports Med..

[7] Järvelä T., Paakkala T., Kannus P., Järvinen M. (2001). The incidence of patellofemoral osteoarthritis and associated findings 7 years after anterior cruciate ligament reconstruction with a bone-patellar tendon-bone autograft.. Am. J. Sports Med..

[8] Williams R.J., Hyman J., Petrigliano F., Rozental T., Wickiewicz T.L. (2004). Anterior cruciate ligament reconstruction with a four-strand hamstring tendon autograft.. J. Bone Joint Surg. Am..

[9] Hart A.J., Buscombe J., Malone A., Dowd G.S. (2005). Assessment of osteoarthritis after reconstruction of the anterior cruciate ligament: A study using single-photon emission computed tomography at ten years.. J. Bone Joint Surg. Br..

[10] Howell SM (1998). Principles for placing the tibial tunnel and avoiding roof impingement during reconstruction of a torn anterior cruciate ligament.. Knee Surg Sports Traumatol Arthrosc.

[11] Kopf S., Pombo M.W., Shen W., Irrgang J.J., Fu F.H. (2011). The ability of 3 different approaches to restore the anatomic anteromedial bundle femoral insertion site during anatomic anterior cruciate ligament reconstruction.. Arthroscopy.

[12] Dargel J., Schmidt-Wiethoff R., Fischer S., Mader K., Koebke J., Schneider T. (2009). Femoral bone tunnel placement using the transtibial tunnel or the anteromedial portal in ACL reconstruction: A radiographic evaluation.. Knee Surg. Sports Traumatol. Arthrosc..

[13] Arnold M.P., Kooloos J., van Kampen A. (2001). Single-incision technique misses the anatomical femoral anterior cruciate ligament insertion: A cadaver study.. Knee Surg. Sports Traumatol. Arthrosc..

[14] Lubowitz J.H., Konicek J. (2010). Anterior cruciate ligament femoral tunnel length: cadaveric analysis comparing anteromedial portal versus outside-in technique.. Arthroscopy.

[15] Lubowitz J.H. (2009). Anteromedial portal technique for the anterior cruciate ligament femoral socket: Pitfalls and solutions.. Arthroscopy.

[16] van Eck C.F., Lesniak B.P., Schreiber V.M., Fu F.H. (2010). Anatomic single and double-bundle anterior cruciate ligament reconstruction flowchart.. Arthroscopy.

[17] Ferraz V., Westerberg P., Brand J.C. (2013). Anterior cruciate ligament femoral socket drilling with a retrograde reamer: Lessons from the learning curve.. Arthrosc. Tech..

[18] Amis A.A., Dawkins G.P. (1991). Functional anatomy of the anterior cruciate ligament. Fibre bundle actions related to ligament replacements and injuries.. J. Bone Joint Surg. Br..

[19] Loh J.C., Fukuda Y., Tsuda E., Steadman R.J., Fu F.H., Woo S.L. (2003). Knee stability and graft function following anterior cruciate ligament reconstruction: Comparison between 11 o’clock and 10 o’clock femoral tunnel placement. 2002 Richard O’Connor Award paper.. Arthroscopy.

[20] Musahl V., Plakseychuk A., VanScyoc A., Sasaki T., Debski R.E., McMahon P.J., Fu F.H. (2005). Varying femoral tunnels between the anatomical footprint and isometric positions: Effect on kinematics of the anterior cruciate ligament-reconstructed knee.. Am. J. Sports Med..

[21] Zavras T.D., Race A., Amis A.A. (2005). The effect of femoral attachment location on anterior cruciate ligament reconstruction: Graft tension patterns and restoration of normal anterior-posterior laxity patterns.. Knee Surg. Sports Traumatol. Arthrosc..

[22] Iriuchishima T., Ingham S.J., Tajima G., Horaguchi T., Saito A., Tokuhashi Y., Van Houten A.H., Aerts M.M., Fu F.H. (2010). Evaluation of the tunnel placement in the anatomical double-bundle ACL reconstruction: A cadaver study.. Knee Surg. Sports Traumatol. Arthrosc..

[23] Lubowitz J.H., Hwang M., Piefer J., Pflugner R. (2014). Anterior cruciate ligament femoral footprint anatomy: Systematic review of the 21^st^ century literature.. Arthroscopy.

[24] Bird J.H., Carmont M.R., Dhillon M., Smith N., Brown C., Thompson P., Spalding T. (2011). Validation of a new technique to determine midbundle femoral tunnel position in anterior cruciate ligament reconstruction using 3-dimensional computed tomography analysis.. Arthroscopy.

[25] Kamath G.V., Redfern J.C., Greis P.E., Burks R.T. (2011). Revision anterior cruciate ligament reconstruction.. Am. J. Sports Med..

[26] Marchant B.G., Noyes F.R., Barber-Westin S.D., Fleckenstein C. (2010). Prevalence of nonanatomical graft placement in a series of failed anterior cruciate ligament reconstructions.. Am. J. Sports Med..

[27] van Eck C.F., Widhalm H., Murawski C., Fu F.H. (2015). Individualized anatomic anterior cruciate ligament reconstruction.. Phys. Sportsmed..

[28] Radermacher K., Portheine F., Anton M., Zimolong A., Kaspers G., Rau G., Staudte H.W. (1998). Computer assisted orthopaedic surgery with image based individual templates.. Clin. Orthop. Relat. Res..

[29] Brown G.A., Milner B., Firoozbakhsh K. (2002). Application of computer-generated stereolithography and interpositioning template in acetabular fractures: A report of eight cases.. J. Orthop. Trauma.

[30] Birnbaum K., Schkommodau E., Decker N., Prescher A., Klapper U., Radermacher K. (2001). Computer-assisted orthopedic surgery with individual templates and comparison to conventional operation method.. Spine.

[31] Hafez M.A., Chelule K.L., Seedhom B.B., Sherman K.P. (2006). Computer-assisted total knee arthroplasty using patient-specific templating.. Clin. Orthop. Relat. Res..

[32] Hananouchi T., Saito M., Koyama T., Hagio K., Murase T., Sugano N., Yoshikawa H. (2009). Tailor-made surgical guide based on rapid prototyping technique for cup insertion in total hip arthroplasty.. Int. J. Med. Robot..

[33] Oka K., Moritomo H., Goto A., Sugamoto K., Yoshikawa H., Murase T. (2008). Corrective osteotomy for malunited intra-articular fracture of the distal radius using a custom-made surgical guide based on three-dimensional computer simulation: case report.. J. Hand Surg. Am..

[34] Murase T., Oka K., Moritomo H., Goto A., Yoshikawa H., Sugamoto K. (2008). Three-dimensional corrective osteotomy of malunited fractures of the upper extremity with use of a computer simulation system.. J. Bone Joint Surg. Am..

[35] Kopf S., Pombo M.W., Szczodry M., Irrgang J.J., Fu F.H. (2011). Size variability of the human anterior cruciate ligament insertion sites.. Am. J. Sports Med..

[36] Araki D., Kuroda R., Kubo S., Fujita N., Tei K., Nishimoto K., Hoshino Y., Matsushita T., Matsumoto T., Nagamune K., Kurosaka M. (2011). A prospective randomised study of anatomical single-bundle *versus* double-bundle anterior cruciate ligament reconstruction: Quantitative evaluation using an electromagnetic measurement system.. Int. Orthop..

[37] Yagi M., Kuroda R., Nagamune K., Yoshiya S., Kurosaka M. (2007). Double-bundle ACL reconstruction can improve rotational stability.. Clin. Orthop. Relat. Res..

[38] Ristanis S., Giakas G., Papageorgiou C.D., Moraiti T., Stergiou N., Georgoulis A.D. (2003). The effects of anterior cruciate ligament reconstruction on tibial rotation during pivoting after descending stairs.. Knee Surg. Sports Traumatol. Arthrosc..

[39] Brandsson S., Karlsson J., Swärd L., Kartus J., Eriksson B.I., Kärrholm J. (2002). Kinematics and laxity of the knee joint after anterior cruciate ligament reconstruction: Pre and postoperative radiostereometric studies.. Am. J. Sports Med..

[40] Logan M., Dunstan E., Robinson J., Williams A., Gedroyc W., Freeman M. (2004). Tibiofemoral kinematics of the anterior cruciate ligament (ACL) deficient weightbearing, living knee employing vertical access open “interventional” multiple resonance imaging.. Am. J. Sports Med..

[41] Tashman S., Collon D., Anderson K., Kolowich P., Anderst W. (2004). Abnormal rotational knee motion during running after anterior cruciate ligament reconstruction.. Am. J. Sports Med..

[42] Tashman S., Kolowich P., Collon D., Anderson K., Anderst W. (2007). Dynamic function of the ACL-reconstructed knee during running.. Clin. Orthop. Relat. Res..

[43] Rabuck S.J., Middleton K.K., Maeda S., Fujimaki Y., Muller B., Araujo P.H., Fu F.H. (2012). Individualized anatomic anterior cruciate ligament reconstruction.. Arthrosc. Tech..

[44] Abebe E.S., Utturkar G.M., Taylor D.C., Spritzer C.E., Kim J.P., Moorman C.T., Garrett W.E., DeFrate L.E. (2011). The effects of femoral graft placement on *in vivo* knee kinematics after anterior cruciate ligament reconstruction.. J. Biomech..

[45] Popescu D., Laptoiu D. (2016). Rapid prototyping for patient-specific surgical orthopaedics guides: A systematic literature review.. Proc. Inst. Mech. Eng. H.

[46] Bundoc R.C., Delgado G.G., Grozman S.A. (2017). A novel patient-specific drill guide template for pedicle screw insertion into the subaxial cervical spine utilizing stereolithographic modelling: An*In Vitro* study.. Asian Spine J..

[47] Cavaignac E., Pailhé R., Laumond G., Murgier J., Reina N., Laffosse J.M., Bérard E., Chiron P. (2015). Evaluation of the accuracy of patient-specific cutting blocks for total knee arthroplasty: A meta-analysis.. Int. Orthop..

